# Obstructive Sleep Apnea Screening in Patients Attending Medical Outpatient Department of Tertiary Care Hospital of Kathmandu: A Cross-sectional Study

**DOI:** 10.31729/jnma.9206

**Published:** 2025-08-31

**Authors:** Bikal Shrestha, Raj Kumar Thapa, Bikash Shrestha, Abhishek Srivastav, Laxmi Rawat

**Affiliations:** 1 Department of Community Medicine, Nepalese Army Institute of Health Sciences, Bhandarkhal, Kathmandu, Nepal; 2 Department of Internal Medicine, Shree Birendra Hospital, Chauni, Kathmandu, Nepal; 3 Intern, MBBS student, Nepalese Army Institute of Health Sciences, Bhandarkhal, Kathmandu, Nepal

**Keywords:** *obstructive sleep apnea*, *polysomnography*, *screening*

## Abstract

**Introduction:**

Obstructive sleep apnea is a common yet underrecognized condition in people marked by upper airway obstruction during sleep, causing fatigue and serious health consequences. Diagnosing obstructive sleep apnea with polysomnography may not be feasible in resource limited-setting, place as in Nepal. Therefore, this study aims to find the prevalence of obstructive sleep apnea among patients using the STOB-BANG questionnaire as a screening tool.

**Methods:**

A cross-sectional survey was conducted among 265 patients attending the medical outpatient department at a tertiary care hospital. The risk of OSA was assessed using a standardised questionnaire, classifying participants into low-risk (score 0-2) and intermediate-to-high risk (score 3-5) groups. A multivariate logistic model was applied to find the risk factors.

**Results:**

Out of 250 patients after exclusion, 183 (73.20%) were age above 50 years and 130 (52%) were hypertensive. Based on the STOP-BANG risk score, 140 (56%; 95% CI: 49.61%-6225%) were categorized as intermediate to high risk and 110 (44%; 95% CI 37.75%-50.39% ) were categorized as low risk of obstructive sleep apnea. Age more than 50 years , male sex, hypertension and diabetes were identified as independent predictors of sleep apnea risk even after adjustment for potential confounders.

**Conclusions:**

The proportion of obstructive sleep apnea was comparably high in our study population.

## INTRODUCTION

Obstructive sleep apnea (OSA) is a complex and multifactorial disease with repetitive apnea and hypopnea.^1^ Global estimates suggest more than 900 million individuals aged 30-69 years were found to have an apnea-hypopnea index (AHI) of five or more and this number may grow with sedentary lifestyles and high-stress environment.^2^,^3^

Although polysomnography (PSG) is gold standard for diagnosis, it is costly and time-consuming. Among the various screening tools for OSA, STOP-BANG is widely used and has a more than 90% pooled sensitivity in severe OSA.^1,4,5^

OSA is highly comorbid, and likned with hypertension (HTN) and resistant HTN (50%), atrial fibrillation (AF) (43-85%), heart failure (HF) (12-53%), chronic kidney disease (CKD) (65%), and obese diabetic (86%).^6-10^ However, OSA risks in these population has not been studied in the context of Nepal. The aims of the study is to find out the prevalence of obstructive sleep apnea in our study population patients using STOP-BANG questionnaire.

## METHODS

This observational study was conducted over a 5 month period from February 2025 to June 2025 at Shree Birendra Hospital (SBH), a 600 bedded tertiary care hospital of Nepal Army that caters treatment services to service personnel and their dependents. Sample size of 265 was calculated based on prevalence 21.5% which was derived from averaging the prevalence of risk of OSA from two similar cross sectional studies taking 5% level of significance and 5% error of margin.^11,12^ The total population considered for the study were all patients visiting medical outpatient department of the hospital. The accessible population were all patients visiting medical outpatient during study period, out of which convenient sampling was done to obtain the sample population. Prevalence of OSA amongst the study population was calculated by dividing total population enrolled in the study by patient with intermediate to high risk OSA. As an inclusion criteria, all patients 18 years and older attending medical outpatient departments were eligible for study and pregnant women and patient with hearing problems were excluded from the study. All eligible patients who signed informed consent were administered the STOP-BANG questionnaire by a well-trained medical interns and medical students who were supervised by the faculties of the same departments until the sample size was met.

The sociodemographic characteristics recorded were: age, sex, education, address and occupation. The comorbidities recorded were hypertension, diabetes mellitus, coronary artery disease, chronic kidney disease, chronic obstructive pulmonary disease, depression and stroke. All the comorbidities were recorded by asking the patients about the disease status and which was further confirmed by checking the treatment card.

The following symptoms were collected subjectively from participants: snoring, apnea witnessed by family members, day time fatigued. Blood pressure (Systolic Blood Pressure= SBP; Diastolic Blood Pressure = DBP), neck circumference, height weight were measure objectively with standardized techniques. Body Mass Index (BMI) was calculated from weight in kg divided by height in m^2^ and a BMI > 30 kg/m^2^ was categorized as obesity. The risk of OSA screened by eight questions that were categorized as “yes” or “no” depending upon the responses and parameters measured. The number of “yes” answers determine the score of the questionnaire. The score between 0-2 was categorized as low risk OSA and the score of 3-5 as intermediate risk and the risk of OSA is high when the score is >5. The STOP-BANG score was further categorized as low risk (0-2) and intermediate to high risk (3-8).^13^ The ethical approval was taken from IRC Institutional Review Committee of Nepalese Army Institution of Health Sciences (Reg. No:1234/Ref.No. 245 dated (12/02/2025) and administrative authorization from commandant of SBH. Subsequently, the written informed consent of all participants were taken during conduction of study. For statistical analysis, data from the questionnaire were manually entered into the Excel sheet and responses were coded as per the requirements of analysis. The final Excel sheet was exported to STATA/SE 17.0 and analysis was done. The categorical data were expressed as frequency and percentage and quantitative data as median and inter quartile range (IQR). The data was further evaluated with multivariate logistic regression taking gender, age groups, education, address category, neck circumference, BMI cut offs, hypertension, diabetes mellitus, COPD, dyslipidaemia, stroke and coronary artery disease (CAD) as independent variables and OSA risk as dependent variable. The multivariate logistic regression was considered as a part of post-hoc analysis. The p-value of less than 0.05 was taken as significant and presented with odds ratio and 95% CI. The Post Hoc analysis was done to find out the power of individual risk factors.

## RESULTS

Of the 265 participants initially enrolled, 15 were excluded as a result of incomplete questionnaire. From the findings of STOP-BANG questionnaire, 183 (73.20%) were above the age of 50 years, 130 (52%) had hypertension and only 8 (3.20%) had neck circumference more than 40 cm. Based on the STOP-BANG risk score, 140 participants (56%; 95% CI: 49.8-62.2%) were categorized as intermediate to high risk and 110 participants (44%; 95% CI: 37.8-50.2%) were categorized as low risk of OSA (Table 1).

**Table 1:** Findings of STOP-BANG questionnaire (n=250).

Variables	n(%)
SNORE loudly	95(38)
Often feel TIRED, fatigue, or sleep during daytime	115(46)
Observed stop breathing during your sleep	60(24)
High blood Pressure or treated for HTN	130(52)
BMI more than 35 kg/m2	16(6.40)
Age over 50 years old	183(73.20)
Neck circumference >16 inches (40 cm)	8(3.20)
Gender: Male	111(44)
Low risk of OSA (0-2) score	110(44)
Intermediate to high risk OSA (3-8) score	140(56)

BMI=Body Mass Index; HTN=Hypertension; OSA=Obstructive Sleep Apnea

The median age of participants was 59.5 years (IQR: 50-66 years). In the high-risk group, the median age was 63 years (IQR: 56-70 years), whereas in the low-risk group it was 52.5 years (IQR: 40-63 years) (Table 2). The median BMI, SBP, DBP, and neck circumference were 25 kg/m^2^ (IQR: 23.2-29.4 kg/m^2^), 120 mmHg (IQR: 120-130 mmHg), 80 mmHg (IQR: 80-80 mmHg), and 36 cm (IQR: 34-38 cm), respectively (Table 2).

A total of 183 participants (73.2%) were above 50 years of age; among them, 126 (68.2%) were at intermediate to high risk and 57 (31.1%) at low risk of OSA. Among males, 80 (72.1%) were at intermediate to high risk. Similarly, intermediate to high risk of OSA was observed in 66 (59.5%) illiterate participants, 99 (76.2%) with hypertension, 55 (70.5%) with diabetes, 24 (80.0%) with COPD, 4 (50.0%) with stroke, and 135 (56.0%) with CAD (Table 3).

Post-hoc multivariate logistic regression showed that sex, age, hypertension, and diabetes were significantly associated with high risk of OSA. Among these, age >50 years had an odds ratio (OR) of 6.62 (95% CI: 3.07-14.30), hypertension OR: 5.87 (95% CI: 3.05-11.30), male sex OR: 4.20 (95% CI: 2.11-8.37), and diabetes mellitus OR: 2.16 (95% CI: 1.06-4.41) (Table 4).

**Table 2:** Physical characteristics of participants based on the STOP-BANG questionnaire grouping (n=250)

Characteristics	Overall (N=250)	Low risk (n=110)	Intermediate to high risk (n=140)
Age in years median(IQR)	59.5(50-66)	52.5(40-65)	63(56-70)
BMI in kg/m2 median(IQR)	25.94(23.19-29.38)	25.94(22.86-29.14)	25.9(23.47-29.79)
SBP (mmHg) median(IQR)	120(120-130)	120(110-130)	120(120-130)
DBP (mmHg) median(IQR)	80(80-80)	80(80-80)	80(78-90)
Neck circumference (cm) 36(34-38)	36(34-37)	36(34-38)

IQR = Interquartile Range; BMI = Body Mass Index; SBP = Systolic Blood Pressure; DBP = Diastolic Blood Pressure

**Table 3:** Characteristics of participants based on the STOP-BANG questionnaire grouping (n=250).

Characteristics		Overall participants (n=250)	Low risk for OSA(0-2) score (n=110)	Intermediate to high risk for OSA (>3) score (n=140)
Age groups (years)	≤ 50	67(26.80)	53(79.10)	14(20.90)
	> 50	183(73.20)	57(31.15)	126(68.15)
Gender	Female	139(55.60)	79(56.83)	60(43.17)
	Male	111(44.60)	31(27.93)	80(72.07)
Address	Kathmandu valley	139(55.60)	65(46.76)	74(53.24)
	Out of valley	111(44.60)	45(40.54)	66(59.46)
Education	Illiterate	64(25.60)	29(45.31)	35(54.69)
	Educated	186(74.40)	81(43.55)	105(56.45)
Hypertension	Yes	130(52)	31(23.85)	99(76.15)
	No	120(48)	79(65.83)	41(34.17)
Diabetes	Yes	78(31.20)	23(29.49)	55(70.51)
	No	172(68.80)	87(50.58)	85(49.52)
COPD	Yes	30(12)	6(20)	24(80)
	No	220(88)	104(47.27)	116(52.73)
Dyslipidemia	Yes	19(7.60)	5(26.32)	14(73.68)
	No	231(92.40)	105(45.45)	126(54.55)
Stroke	Yes	8(3.21)	4(50)	4(50)
	No	241(96.79)	106(43.98)	135(56.02)
CAD	Yes	22(8.80)	10(45.45)	12(54.55)
	No	228(91.20)	100(43.86)	128(56.14)

COPD= Chronic Obstructive Pulmonary Disease, CAD= Coronary Artery Disease; OSA= Obstructive Sleep Apnea

**Table 4:** Risk of OSA categorized by STOP-Bang among all study participants (n=250).

Factors	n(%)	OR(95% CI)^a^	p-value^a^	Estimated Post Hoc Power
Male sex	111(44.40)	4.20(2.11-8.37)	0.001	>95%
Age > 50 years	183(73.20)	6.62(3.07-14.30)	0.001	>99%
Hypertension	130(52)	5.87(3.05-11.30)	0.001	>95%
Diabetes mellitus	78(31.20)	2.16(1.06-4.41)	0.035	~60-70%
Neck circumference >40 cms	69(27.60)	1.35(0.64-2.87)	0.432	<40%
Illiterate	186(74.40)	1.07(0.61-1.90)	0.806	<20%
COPD	30(12)	2.96(0.98-8.96)	0.055	~50-60%
BMI 30 or more	55(22)	1.36(0.74-2.51)	0.326	<20%
Stroke	8(3.21)	0.79(0.19-3.21)	0.737	<20%
Dyslipidemia	19(7.60)	1.84(0.45-7.55)	0.389	<20%
CAD	22(8.80)	0.94(0.39-2.26)	0.886	<20%

a Adjusted; OR = Odds Ratio; CI = Confidence Interval; STOP-Bang = Snoring, Tiredness, Observed Apnea, Hypertension, Body Mass Index, Age, Neck Circumference, and Gender; BMI = Body Mass Index; COPD = Chronic Obstructive Pulmonary Disease; CAD = Coronary Artery Disease

## DISCUSSION

This hospital based sftudy address the risk of sleep apnea in patient attending medical OPD of tertiary care hospital with validated STOP-Bang questionnaire as evidence by previous systematic review. Based on the scoring of STOP-Bang, 44% were categorized as low risk, 52% as intermediate risk, and 4% as high risk of OSA. Several Judies using STOP-Bang questionnaire in Asian population reported prevalence ranging from 12% to 63% of high OSA risk depending upon the patients comorbid status and population representative.^11, 12, 14, 15^ The finding from this study on high risk OSA population was on a bit lower side (4%), compared to other studies.^11^ However, the participants in the intermediate risk group were higher (52%) as compared to 23.33%.^15^ The disparity in the results may be due the participants enrolled with lower comorbidities in terms of diabetes (100% vs 31%) and obesity (55.2% vs 22%).^12^ Therefore, the percentage of people falls in the category of intermediate to high risk of OSA should be aware to prevent adverse consequences.

In our studies, the cut off for neck circumference and BMI were found out to be nonsignificant related with OSA risks. Studies conducted in Asian population regarding BMI and associated risk revealed that risk of non-communicable diseases like diabetes and hypertension started at BMI less than 25 kg/m^2^ which is stated normal for western population.^16^ Since the risk categorization was developed in and for the western population, cut off parameters of factor like BMI should be lowered down for ur population. Moreover, the post hoc power analysis revealed that the power estimates based on the result of our study for BMI category were less than 20% which signify further research with larger sample size.

Study revealed that the male proportion are significantly higher (57.14%) in high risk group compared to female (42.86%). The prevalence of high risk of OSA in males, compared with females which has been persistently demonstrated as a risk factor for OSA from the previous epidemiological studies.^11, 12^ Although our study demonstrated the male proportion was predominant in high risk OSA but the distribution of other characteristics among the genders were not largely different and this finding is comparable with other studies as well.^11^

Out of eight questions of the STOP-BANG questionnaire, the most common observed risk item was age above 50 years (73.2%) which was followed by hypertension (52%), tiredness (46%) and male gender (44%) respectively. The neck circumference above 40 cm was the lowest item observed among study participants. In our study, higher STOP-BANG score was associated with older adults males, hypertension and diabetes even after adjusting other variables. This finding was consistent with other studies.^6, 11, 17^ However, as this observation was found in post-hoc analysis there is a chance of type I error . Nevertheless, OSA is already a proven risk factor cardiovascular diseases, particularly hypertension, diabetes and stroke which are further exacerbated by obesity.^11^

The intermittent hypoxia and sleep fragmentation in OSA patients increases sympathetic and Renin-Angiotensin-Aldosterone System activation that raises blood pressure by vasoconstriction and blood volume increment.^1^ Furthermore, it increases the diabetes mellitus by affecting plasma insulin level and glycemia.^18^ Based on these results the male patient above the age of 50 years with diabetes and hypertension should be screened in order to decrease the impact from chronic metabolic diseases. Some of the risk factors like neck circumference, education category and obesity are significantly associated with risk of OSA in other studies which may be due to larger sample size and more strict inclusion criteria for study population.^11,12^ However, the proportion of mentioned risk factors in our study were slightly on the lower side and also post hoc power calculated based on the findings demonstrated the estimated power < 70%. In order to check for the association, the further study with larger sample size needs to be carried out to mitigate the possible type II error.

There are some limitations in the present study, first these patients were not further investigated with polysomnography to confirm to have OSA but reviews show that STOP-BANG has both good sensitivity and specificity in comparison to PSG. The risk categorization was solely based on the participant’s response. However, the fact revealed from the study was similar to the study conducted in similar setting. Second, since the study was a hospital based, there might be selection bias in the participant but the hospital selected was one of the tertiary care center where the patients come from all over Nepal including all geographical distribution. Finally, study was cross-sectional design where causal association between high risk of OSA and other comorbidities and their medications cannot be ascertained. This study is the first of its kind to report the prevalence of risk of OSA based on the STOP-Bang questionnaire in the patient attending to one of the tertiary care center of Kathmandu and provides the awareness of risk of OSA in comorbid patients.

## CONCLUSIONS

The risk of OSA in our study population was comparatively high; OSA must be screened and detected early in elderly male above 50 years with hypertension and diabetes mellitus in order to reduce impact from clinical consequences of OSA.

## Data Availability

The data are available from the corresponding author upon reasonable request.

## References

[1] Chang JL, Goldberg AN, Alt JA, Mohammed A, Ashbrook L, Auckley D (2023). International Consensus Statement on Obstructive Sleep Apnea.. Int Forum Allergy Rhinol..

[2] Benjafield AV, Ayas NT, Eastwood PR, Heinzer R, Ip MSM, Morrell MJ (2019). Estimation of the Global Prevalence and Burden of Obstructive Sleep Apnoea: A Literature-Based Analysis.. Lancet Respir Med..

[3] De Batlle J, Gracia-Lavedán E, Escarrabill J, García-Altés A, Martinez Carbonell E, Henríquez-Beltrán M (2024). Effect of CPAP Treatment on Cardiovascular Outcomes.. Arch Bronconeumol..

[4] Patel D, Tsang J, Saripella A, Nagappa M, Islam S, Englesakis M (2022). Validation of the STOP Questionnaire as a Screening Tool for OSA Among Different Populations: A Systematic Review and Meta-Regression Analysis.. J Clin Sleep Med..

[5] Chiu HY, Chen PY, Chuang LP, Chen NH, Tu YK, Hsieh YJ (2017). Diagnostic Accuracy of the Berlin Questionnaire, STOP-BANG, STOP, and Epworth Sleepiness Scale in Detecting Obstructive Sleep Apnea: A Bivariate Meta-Analysis.. Sleep Med Rev..

[6] Drager LF, Genta PR, Pedrosa RP, Nerbass FB, Gonzaga CC, Krieger EM (2010). Characteristics and Predictors of Obstructive Sleep Apnea in Patients with Systemic Hypertension.. Am J Cardiol..

[7] Moula AI, Parrini I, Tetta C, Lucà F, Parise G, Rao CM (2022). Obstructive Sleep Apnea and Atrial Fibrillation.. J Clin Med..

[8] Polecka A, Olszewska N, Danielski Ł, Olszewska E (2023). Association between Obstructive Sleep Apnea and Heart Failure in Adults-a Systematic Review.. J Clin Med..

[9] Li X, Liu C, Zhang H, Zhang J, Zhao M, Sun D (2019). Effect of 12-Month Nasal Continuous Positive Airway Pressure Therapy for Obstructive Sleep Apnea on Progression of Chronic Kidney Disease.. Medicine (Baltimore)..

[10] Kline CE, Reboussin DM, Foster GD, Rice TB, Strotmeyer ES, Jakicic JM (2016). The Effect of Changes in Cardiorespiratory Fitness and Weight on Obstructive Sleep Apnea Severity in Overweight Adults with Type 2 Diabetes.. Sleep..

[11] Lee SY, Yu H, Kim DK (2022). Glaucoma Is Associated with the Risk of Obstructive Sleep Apnea: A Population-Based Nationwide Cohort Study.. Diagnostics (Basel)..

[12] Al-Sebaei MO, Bamashmous MS, Mirdad MH, Sindi MA (2023). Prevalence of Patients at Risk for Developing Obstructive Sleep Apnea Based on the STOP-BANG Questionnaire at King Abdulaziz University Faculty of Dentistry.. Clin Exp Dent Res...

[13] Shnaimer JA, Dahlan HM, Hanbashi FM, Bahammam AS, Gosadi IM (2022). Assessment of the Risk of Obstructive Sleep Apnoea Among Patients with Type 2 Diabetes and Its Associated Factors Using the STOP-BANG Questionnaire: A Cross-Sectional Study.. J Taibah Univ Med Sci..

[14] Chung F, Abdullah HR, Liao P (2016). STOP-BANG Questionnaire.. Chest..

[15] Sajjanar J, Johari S, Gade J, Kumbhalwar A, Deshpande I, Soni M (2021). Determining the Risk of Obstructive Sleep Apnea (OSA) Using STOP-BANG Questionnaire: An Observational Study.. Ann Rom Soc Cell Biol...

[16] Goh VH, Tain C, Tong TY, Mok HP, Wong M (2004). Are BMI and Other Anthropometric Measures Appropriate as Indices for Obesity? A Study in an Asian Population.. J Lipid Res..

[17] Kang J, Koo HK, Kang HK, Seo WJ, Kang J, Kim J (2024). Prevalence of High-Risk Group for Obstructive Sleep Apnea Using the STOPBANG Questionnaire and Its Association with Cardiovascular Morbidity.. Front Neurol..

[18] Elmasry A, Lindberg E, Berne C, Janson C, Gislason T, Tageldin MA (2001). Sleep-disordered Breathing and Glucose Metabolism in Hypertensive Men: A Population-based Study.. J Intern Med..

